# Factors associated with tooth wear in a Swedish adult population: a cross‑sectional study

**DOI:** 10.1186/s12903-026-09417-w

**Published:** 2026-07-25

**Authors:** Martin Ågren, Catharina Österlund, Mats Sjöström, Anastasios Grigoriadis, Ola Norderyd, Bo Rolander, Mattias Pettersson

**Affiliations:** 1 Department of Odontology, Faculty of Medicine, Umeå University, Umeå, 901 87 Sweden; 2 Industrial Doctoral School for Research and Innovation, Umeå University, Umeå, Sweden; 3 Department of Dental Medicine, Karolinska Institutet, Stockholm, Sweden; 4 Department of Odontology and Oral Health, Jönköping University, Jönköping, Sweden; 5 Futurum, Academy for Health and Care, Region Jönköping County, Jönköping, Sweden

**Keywords:** Tooth wear, Attrition, Abrasion, Erosion, Bruxism

## Abstract

**Background:**

Tooth wear encompasses the loss of dental hard tissues through processes including attrition, abrasion and erosion. Previous studies have identified potential associations with age, sex, salivary characteristics, socioeconomic factors and bruxism; however, findings remain inconsistent and often based on single-factor analyses. This study aimed to investigate factors associated with tooth wear, with emphasis on subject‑based bruxism, and to evaluate the relative contribution of bruxism in relation to demographic, socioeconomic, clinical, and functional factors using a multivariate analytical approach.

**Methods:**

Data were derived from cross-sectional dental surveys and examinations conducted in Jönköping, Sweden. Participants aged 20 to 80 years underwent standardised clinical examinations and completed questionnaires addressing oral health, symptoms, behaviours and sociodemographic factors. Tooth wear was recorded separately as erosive or physical wear (attrition or abrasion) and was also combined to represent overall tooth wear. Bruxism was assessed by subject-based self-report, and temporomandibular disorder was evaluated using the Helkimo Clinical Dysfunction Index. Stimulated salivary flow rate and buffer capacity were measured. Associations between potential explanatory factors and tooth wear were analysed using generalised linear models, and between subjective assessments and tooth wear by Spearman rank correlations.

**Results:**

Among 909 eligible dentate participants with adequate occlusal support, 646 had complete datasets for multivariable modelling. Physical tooth wear showed near linear correlation with overall tooth wear, whereas association between overall tooth wear and erosive wear was moderate. In adjusted analyses, subject-based bruxism, higher age, and male sex were independently associated with a higher proportion of teeth affected by tooth wear. Tooth wear was also significantly associated with reduced self-perceived chewing capacity and aesthetics. No significant associations were observed for educational level, body mass index, number of remaining teeth, temporomandibular disorder, salivary flow rate, buffer capacity, or medical-condition–related attendance.

**Conclusions:**

In this Swedish population, tooth wear was more strongly associated with physical loading than with erosive processes. Subject-based bruxism showed the strongest independent association with tooth wear, exceeding the impact of demographic factors. These findings highlight the clinical relevance of patient-reported clenching or grinding when evaluating tooth wear and support its potential role in identifying individuals with increased levels of tooth wear.

**Supplementary Information:**

The online version contains supplementary material available at 10.1186/s12903-026-09417-w.

## Background

Tooth wear is a common clinical finding in adults and may lead to dental sensitivity, impaired oral function, and the need for extensive restorative treatment [1, 2]. Multiple factors have been proposed to contribute to tooth wear, including higher age, male sex, lower education level, hyposalivation, dietary habits, diseases, higher body mass index (BMI) and bruxism [3]. However, previous studies have reported inconsistent results and have relied largely on single‑factor analyses, highlighting the need for multivariable approaches to clarify independent associations [3]. This uncertainty complicates clinical decision-making, as tooth wear is often identified at a late stage and preventive strategies are applied inconsistently due to limited guidance on the most influential risk factors in adult populations [4, 5].

Behaviours related to functional and parafunctional loading, such as grinding of the teeth, which can be a manifestation of bruxism, are mechanistically plausible contributors to tooth wear but are not routinely assessed in clinical practice [6, 7]. However, despite long ongoing research, it remains unclear whether bruxism is independently associated with tooth wear after adjustment for established confounding factors such as age, sex, socioeconomic status, salivary characteristics, and remaining teeth [3]. Previous studies have reported conflicting findings, often due to differences in bruxism definitions in and between disciplines of odontology and medicine, limited adjustment for confounders, or inclusion of selected populations rather than representative adult cohorts [8–10]. This uncertainty has contributed to the ongoing debate regarding the clinical importance of bruxism in the development and progression of tooth wear. Knowledge on bruxism has rapidly been evolving as international consensus meetings since the early 2010 s have addressed this issue and have introduced the Standardised Tool for the Assessment of Bruxism (STAB) and proposed three parallel assessment approaches with no hierarchy implied; subject‑based, clinically based, and device‑based [10–12].

Clarifying the role of bruxism in tooth wear requires population-based studies that are sufficiently large, representative, and analytically robust [10]. Although device-based assessments such as electromyography or polysomnography provide detailed recordings of jaw muscle activity, they previously have rarely been feasible in epidemiological studies or routine dental practice [10, 13]. Consequently, subject-based assessment of bruxism, typically using self-reported questions on clenching or grinding, represents the most accessible approach in everyday routine practice [10, 13]. Determining whether such pragmatic measure is meaningfully associated with clinical tooth wear is therefore essential for translating research findings into preventive and clinical strategies [7, 10, 11].

The dental epidemiological Jönköping study is a long-standing population-based investigation performed decennially since 1973, with randomly selected adult cohorts aged 20 to 80 years [14]. The municipality of Jönköping is demographically representative of the Swedish population, providing a unique opportunity to investigate tooth wear and associated factors in a large, representative adult sample. While tooth wear data from the Jönköping study have been reported previously, analyses have been limited to the 1983 survey [15, 16]. Although dental epidemiological data on caries prevalence and number of remaining teeth from the 2023 survey are not yet published, several oral health indicators in Sweden have plateaued at relatively low disease levels since the early 2010 s [17]. Moreover, although temporomandibular disorders (TMD) and orofacial pain have been documented in this cohort, their potential overlap with subject‑based bruxism and relationship to tooth wear have not been examined [18]. Validated tools such as the Helkimo Clinical Dysfunction Index (HCDI) facilitate differentiation between pain‑related conditions and loading‑related behaviours [19, 20].

Importantly, few population-based studies have examined whether subject-based bruxism remains associated with tooth wear after simultaneous adjustment for multiple established risk factors, and many studies have included predominantly young and female cohorts [8]. As a result, the independent contribution of bruxism to tooth wear remains insufficiently understood. Addressing these limitations by applying contemporary bruxism definitions and robust multivariable analyses in a representative adult population is therefore warranted.

## Methods

### Aim

The primary aim of this study was to investigate factors independently associated with tooth wear in a representative adult population in Sweden. The secondary aim was to evaluate the relative contribution of subject-based bruxism in relation to demographic, socioeconomic, clinical, and functional factors associated with tooth wear using a multivariate analytic approach.

### Participants

This study is part of the longitudinal Jönköping study, conducted by Jönköping University in collaboration with the Institute for Postgraduate Dental Education in Region Jönköping County, Sweden. Randomly selected adults aged 20 to 80 years were invited to participate in a dental examination. Invitations were sent by mail and complemented by telephone contact when possible. Participants were drawn from the general population of Jönköping, and the present study includes individuals examined in 2013 and 2023. All invited individuals were residents of the registration districts, or their predecessors with unchanged geographical borders, roughly encompassing the town of Jönköping but excluding the districts of the formerly incorporated town of Huskvarna.

Initially, 130 individuals were randomly selected from each of the age group, 20, 30, 40, 50, 60, 70, and 80 years, and invited to participate. Additional randomised individuals were subsequently invited to compensate for non-respondents within the respective age groups.

### Ethics approval and consent to participate

The study was approved by the relevant ethical review bodies at the time of data collection: The 2013 data collection was approved by the Regional Ethical Review Board at Linköping University, Linköping, Sweden (Reference No. 2012/191–31), which at that time was responsible for ethical review of human research conducted in Jönköping County. The 2023 data collection was approved by the Swedish Ethical Review Authority (reference number 2022–04308-01). As no clinical intervention was performed, clinical trial registration was not applicable. The study was conducted in accordance with the Declaration of Helsinki at all time points, and written informed consent was obtained from all participants prior to inclusion in the study.

### Materials

The study protocol has been kept as consistent as possible since 1973 to ensure reliable longitudinal comparisons. Consequently, the study questionnaire and clinical examination protocols used in the two sessions included in the present analysis were identical regarding analysed variables. Salivary buffer capacity was assessed using a chair-side buffer capacity test (Dentobuff Strip, Orion Diagnostica, Espoo, Finland).

### Procedure

#### Questionnaire

After accepting the invitation, the participants completed either a digital or paper-based questionnaire. The questionnaire covered sociodemographic factors, general and oral health, oral behaviours and symptoms, self-reported bruxism, subjective assessments of tooth appearance (0–10 scale) and self-perceived chewing ability (4-point scale). Educational attainment was recorded in an ordinal scale and categorised as compulsory education, upper secondary education, and academic degree.

#### Clinical examination

A standardised clinical examination lasting approximately 60 to 90 min was performed for every study participant. Although examiner calibration was performed, formal reliability statistics were not calculated. The clinical examinations in 2013 were performed by eight dentists from the Institute for Postgraduate Dental Education, and by three general practitioners from the Public Dental Health Service, in Jönköping, Sweden. In 2023 the examinations were performed by seven dentists of which five from the Institute for Postgraduate Dental Education, and two general practitioners from the Public Dental Health Service in Jönköping, Sweden. All dentists from the Institute for Postgraduate Dental Education in the 2023 examinations had also participated in the 2013 study, thereby contributing to methodological continuity and consistency in clinical assessment across the two examination periods. The examination protocol included assessment of tooth wear, registration of occlusal support zones using the Eichner Index [21], examination for signs of TMD using HCDI [20] and recording of the number of remaining teeth. Stimulated salivation flow was measured by chewing on a 1-g piece of paraffin for 3 min, during which saliva was continuously collected in a graded test-tube and then analysed for buffer capacity.

#### Diagnostic criteria

**Fig. 1:**
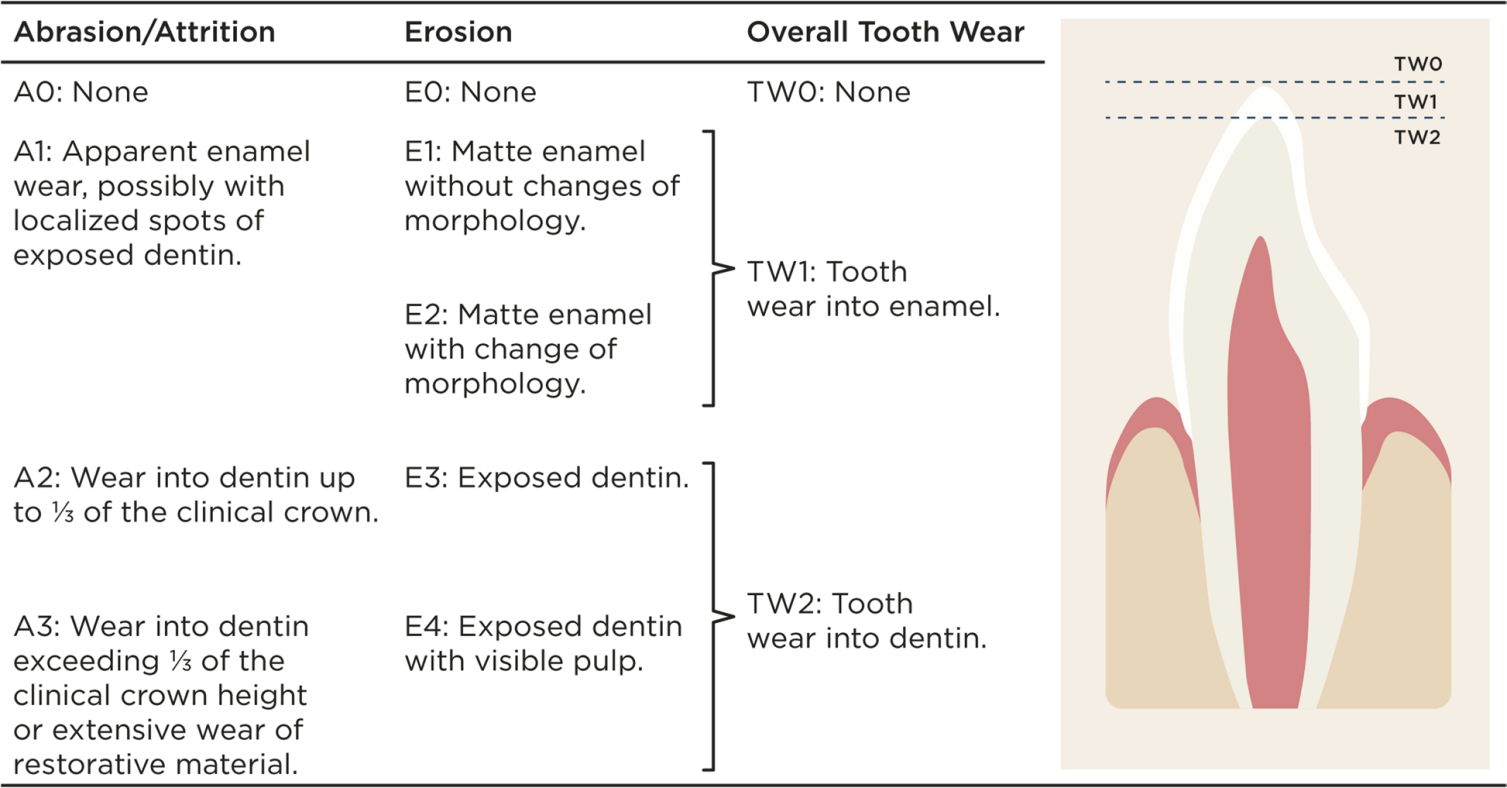
Classification system for tooth wear used in the analysis. Illustration by Marlene Lahti, Inhousebyrån, Umeå University

Physical tooth wear (attrition and abrasion) and erosive tooth wear were recorded separately (Fig. 1), and are in accordance with the definition of tooth wear adopted by the World Dental Federation General Assembly (FDI) [22]. Third molars were excluded from all assessments, resulting in a maximum of 28 teeth per individual. For analyses of overall tooth wear, physical and erosive wear, categories were pooled into a single composite tooth wear variable (Fig. 1). For each tooth, the overall tooth wear score was defined by the most severe value observed, such that the higher of the converted erosive or attrition/abrasion scores determined the composite tooth wear score. The proportion of worn teeth was expressed as a continuous variable between 0 and 1, where 1 indicated the presence of tooth wear on all remaining teeth.

Subject-based bruxism was defined by a positive answer (“Yes, I do”) to the question *“Do you clench or grind your teeth?”*, which partly covers section A1 and A2 of the STAB regarding subject-based assessment of bruxism [23]. The negative option was “No, not that I am aware of”. Individuals lacking more than two occlusal support zones according to the Eichner index were excluded from the analyses [21]. Hyposalivation and low saliva buffer capacity were dichotomized using established cut-offs, defined as a stimulated salivary flow rate of ≤ 0.7 mL/minute, and buffer capacity pH < 5.5, respectively [24]. TMD were screened using HCDI, which is categorised into four levels (0 = no dysfunction, 1 = mild dysfunction, 2 = moderate dysfunction and 3 = severe dysfunction) [20].

### Statistical analyses

Participants with missing data (excluding subjective outcome data) in any of the variables included in the multivariable model were excluded using complete-case analysis. Multiple imputation was considered; however, exclusion primarily arose from incomplete questionnaire responses, where suitable auxiliary information for reliable imputation was limited. Complete-case analysis was therefore selected to maximise transparency and interpretability of the analyses. The characteristics of the studied groups were analysed through descriptive statistics. The relationship between erosive and physical tooth wear to overall tooth wear were analysed through Pearson correlation coefficient. Associations between potential predictors and the proportion of tooth wear were examined using generalised linear models (GLMs) with a Gaussian distribution and identity link function. Although the outcomes were expressed as proportions bounded between 0 and 1, the primary outcome of tooth wear was derived from the proportion of affected teeth and displayed a broad distribution across the interval. Gaussian models were selected because they provided clinically interpretable coefficient estimates representing absolute differences in the proportion of affected teeth. Alternative approaches such as beta regression were considered; however, the dentin-related outcomes contained a substantial proportion of exact zero values, requiring more complex zero-inflated modelling approaches. Model fit was therefore assessed through residual diagnostics and goodness-of-fit statistics. Parameters were estimated using maximum likelihood estimation. Robust covariance estimates were used to obtain standard errors, while variable significance was assessed using Type III likelihood-ratio χ^2^ tests. Confidence intervals were derived using profile likelihood estimation.

Model fit was evaluated using deviance and Pearson chi-square statistics, information criteria (Akaike's and Bayesian), omnibus likelihood-ratio tests, and examination of Pearson residuals. Associations between subjective assessments on performance of chewing and perceived aesthetics and tooth wear were evaluated using Spearman rank correlation coefficients. Owing to the ordinal nature of the subjective outcome variables, Spearman's rho was used to assess the strength and direction of the associations. Data analyses were performed using IBM SPSS Statistics (version 30.0; IBM, Armonk, NY, USA) software. The study sample was determined by a predefined population-based cohort rather than by a sample-size calculation. All statistical tests were two-sided, and *p* < 0.05 was considered statistically significant.

## Results

### Study population

**Fig. 2:**
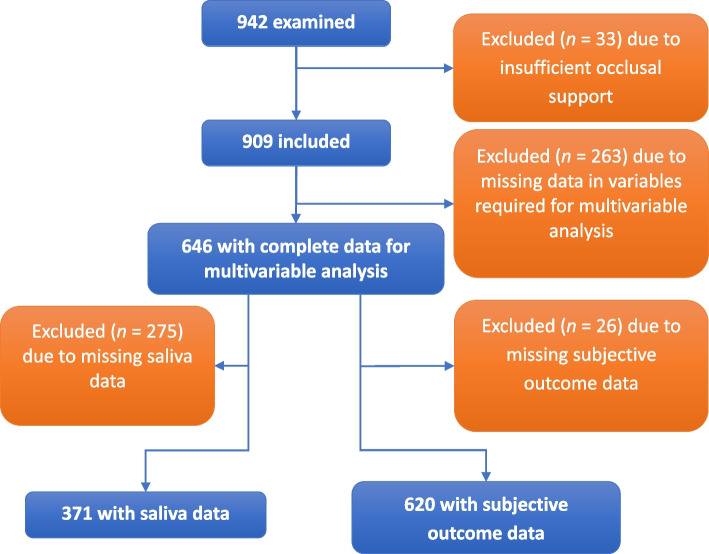
Flowchart of study participants and inclusion in analytical samples

A total of 942 participants were examined (Fig. 2). After excluding edentulous individuals (*n* = 9) and those lacking more than 2 supportive occlusal zone according to the Eichner index (*n* = 24), 909 participants were eligible for inclusion. Complete data for all variables required in GLM were available for 646 participants (Table 1), as some did not complete the digital or printed questionnaire. Saliva data was available for 371 out of the 646 participants, all from the 2013 session as collection of salivary data was discontinued after 2013. Out of the 646 participants 26 missed subjective outcome data.

**Table 1:** Number of study subjects by age group and sex

Age in years	Women	Men	Total
20	64	28	92
30	32	33	65
40	36	43	79
50	48	32	80
60	59	61	120
70	67	61	128
80	40	42	82
*Total*	*346*	*300*	*646*

The mean age of included participants was 52 years. The statistically average participant had about 25 teeth, with at least seven of them affected by wear, including at least one tooth worn into dentin (Table 2). Out of the 646 participants, 131 were categorised with subject-based bruxism, representing 20.3% of all the participants. Inclusion of salivary variables in the multivariable analysis substantially reduced the sample size (*n* = 371) and resulted in only minor alterations of the pattern of significant predictors (Table 3; Additional Table 1). Therefore, salivary variables were excluded from the final model. No pontic was classified as abraded (Attrition/Abrasion grade 3) and thus did not influence the calculated tooth wear proportions.

**Table 2:** Descriptive statistics of the study variables

**Variable** *N* = 646	**Mean (± SD)**
Age in years	52.12 (± 19.72)
Number of teeth	25.69 (± 3.49)
Proportion of overall tooth wear	0.28 (± 0.24)
Proportion of tooth wear into dentin	0.04 (± 0.11)

**Table 3:** Generalised linear model analysis of included variables

Variable	Overall tooth wear		Tooth wear into dentin	
*N* = 646	***P* value**	**Coefficient (95% confidence interval)**	***P* value**	**Coefficient (95% confidence interval)**
Male sex	0.004	0.055 (0.018–0.092)	0.026	0.019 (0.002–0.036)
Higher age	0.002	0.002 (0.001–0.003)	0.058	0.000 (0.000–0.001)
More teeth	0.769	0.001 (−0.005 – 0.007)	0.110	−0.002 (−0.005 – 0.000)
Higher body mass index	0.191	0.003 (−0.001 – 0.007)	0.007	0.003 (0.001–0.004)
Subject-based bruxism	< 0.001	0.117 (0.071–0.162)	< 0.001	0.044 (0.024–0.065)
Higher Helkimo clinical dysfunction index (reference category 3)	0.490	0: −0.083 (−0.201 – 0.036) 1: −0.091 (−0.207 – 0.025) 2: −0.083 (−0.204 – 0.038)	0.199	0: −0.011 (−0.064 – 0.043) 1: −0.002 (−0.054 – 0.051) 2; −0.026 (−0.080 – 0.029)
Higher attained educational level (reference category academic degree)	0.518	CS: −0.029 (−0.082 – 0.025) SS: −0.001 (−0.042 – 0.040)	0.278	CS: 0.006 (−0.018 – 0.030) SS: 0.015 (−0.004 – 0.033)
More regular medical check-ups	0.058	−0.019 (−0.072 – 0.034)	0.610	0.012 (−0.012 – 0.036)

*P* values were obtained using Type III likelihood‑ratio (LR) χ^2^ tests, representing partial effects of each variable adjusted for all other covariates in the multivariable model. CS = Compulsory school. SS = Secondary school

### Correlations between types of tooth wear

Pearson correlation analysis showed a moderate association between overall tooth wear and erosive wear, whereas physical tooth wear demonstrated a strong, near linear correlation with overall tooth wear. A similar pattern was observed for wear into dentin: physical wear into dentin showed a strong correlation with overall dentin wear, while erosive dentin wear was only moderately correlated (Table 4) [25]. The influence of salivary variables on these correlations can be found in Additional Table 2.

**Table 4:** Pearson correlation between the different types of tooth wear

**Degree of tooth wear** *N* = 646	**Classification of tooth wear**	**Pearson correlation**	***P* value**
Overall tooth wear	Due to attrition or abrasion	0.953	< 0.001
	Due to erosion	0.475	< 0.001
Tooth wear into dentin	Due to attrition or abrasion into dentin	0.899	< 0.001
	Due to erosion into dentin	0.467	< 0.001

### Predictors of tooth wear

In the multivariable GLM, self-reported bruxism, higher age, and male sex were independently associated with a greater proportion of tooth wear (all *p* < 0.01). Bruxism showed the strongest effect, followed by age. No significant associations were found for BMI, number of remaining teeth, educational level, HCDI, or healthcare visits due to medical conditions (Table 3).

### Subjective assessments

For analyses of subjective outcomes, 26 participants with missing responses regarding tooth shape and chewing ability were excluded, leaving 620 individuals for the Spearman rank correlation analyses. Greater levels of tooth wear were weakly but significantly associated with lower satisfaction with tooth shape and reduced self-perceived chewing ability (Table 5).

**Table 5:** Associations between subjective assessments and tooth wear (Spearman rank correlations)

*N* = 620	**Subjective assessment**	**Spearman rho**	***P* value**
Overall tooth wear	Shape of teeth (scale of 10)	−0.102	0.011
	Performance of chewing (scale of 4)	−0.146	< 0.001
Tooth wear into dentin	Shape of teeth (scale of 10)	−0.098	0.014
	Performance of chewing (scale of 4)	−0.117	0.003

## Discussion

The main finding of this study is that subject-based bruxism showed the strongest independent association with tooth wear, both for overall wear and for wear extending into dentin. From a clinical perspective, early and effective identification of individuals at risk is desirable as tooth wear has often been overlooked in clinical practice, even among those with advanced wear [26]. Routine assessment during regular dental examinations may help identify patients who, relative to their age and sex, exhibit pronounced tooth wear as early detection and preventive intervention may reduce the future need for extensive restorative treatment. Similar screening approaches have previously been recommended in the context of temporomandibular disorders, where routine questioning may facilitate identification and management of affected individuals [27]. However, the observed association should be interpreted with caution.

Although subject-based bruxism was strongly associated with tooth wear, the possibility of reverse causality must be considered. Individuals with extensive tooth wear may previously have been informed that they grind or clench their teeth and therefore be more likely to report such behaviours. An example of potential reverse causality is that individuals using occlusal splints have been reported to exhibit more tooth wear than those not using splints [28], most likely because splints are more often prescribed to individuals with tooth wear. Thus, reverse causality may partly explain the observed association, a limitation inherent to cross-sectional questionnaire-based studies [8]. Further research is needed to determine whether suggested screening strategies improve clinical outcomes.

Age and sex were statistically robust predictors; however, their effect sizes were relatively modest, especially for age, compared to subject-based bruxism. The coefficient associated with self-reported clenching or grinding (subject-based bruxism) corresponded to an increase in tooth wear that exceeded the estimated effect of several decades of ageing, highlighting that the clinical relevance of subject-based bruxism may be greater than suggested by statistical significance testing alone. This observation may help explain findings from a radiographic study in which the mean loss of anatomical crown length of the central incisors between 25 and 70 years of age was approximately 1 mm in both jaws, while around one fifth of the participants exhibited a loss exceeding 2 mm [29]. Notably, a similar proportion of participants in the present study reported subject-based bruxism, suggesting that parafunctional loading behaviours may contribute to the considerable inter-individual variation in tooth wear. The finding of subject-based bruxism highlights the importance of functional loading behaviours in the development of tooth wear and this complements previous studies reporting inconsistent associations when bruxism was omitted or insufficiently defined [3, 8]. In several earlier studies, bruxism has been defined solely from the presence of tooth wear or temporomandibular pain, which limits causal interpretation [8, 30–32]. Such definitions do not, however, explain the observed sex differences. Several explanations have been suggested for the greater vulnerability observed in men, including histological differences such as thinner enamel thickness and lower degree of prism organisation, greater jaw muscle strength, and behavioural factors, including higher consumption of acidic beverages or foods [33–36].

The pattern of significantly associated predictors observed in this study is consistent with findings from previous Swedish population-based studies [15, 16, 26]. A distinguishing feature of the present study is the inclusion of at least eight potential predictors (ten when salivary variables were considered), providing a more comprehensive multivariable assessment than many earlier studies [3]. Another strength is the broad age range and inclusion of both sexes without restriction to specific occupational or clinical subgroups.

The absence of formal inter‑examiner reliability statistics represents an important limitation, as the assessment of tooth wear involves a degree of subjective judgement. This limits the ability to quantify diagnostic agreement between examiners and may introduce measurement variability. Although examiner calibration was performed prior to data collection, the lack of statistical measures of agreement prevents a formal evaluation of consistency across examiners. Nevertheless, several factors likely mitigated this limitation. Standardised diagnostic criteria were applied throughout the study, and both examination periods were conducted under the same principal investigator. In addition, there was substantial overlap in the examiner cohort, with the majority of the same dentists participating in both examination periods. This continuity, together with calibration procedures, likely contributed to reducing inter‑examiner variability, although it cannot be quantified.

Another limitation is the use of complete-case analysis, which resulted in exclusion of 263 otherwise eligible participants. If individuals with missing data differed systematically from those included in the analyses, selection bias may have been introduced. The missing data was not formally assessed, and it therefore cannot be determined whether data was missing completely at random. Consequently, some risk of selection bias remains.

Although the tooth wear outcomes were expressed as proportions, the overall tooth wear outcome showed a broad distribution across the interval, while dentin wear outcomes contained many exact zero values. The Gaussian identity-link model was selected because the primary outcome displayed a broad distribution across the interval and because the resulting coefficients can be interpreted directly as absolute differences in the proportion of affected teeth. Alternative approaches such as beta regression were considered; however, the dentin-related outcomes contained a substantial proportion of exact zero values, requiring more complex modelling approaches. Future studies may evaluate alternative modelling approaches for proportional outcomes.

The subject-based definition of bruxism used in this study does not distinguish between muscle activity associated to pain or related to tooth wear. While screening tools for TMD are commonly used in general dental practice [37], none of the validated short forms includes a specific question on subject-based assessment of bruxism [38]. Although TMD and bruxism often coexist, their interrelationship remains debated [8, 32]. In the present cohort, no association was observed between HCDI scores and tooth wear, suggesting a lack of correlation between TMD-related pain and tooth wear. Previous data reporting higher HCDI scores among individuals with subject-based bruxism [16], indicates that distinguishing muscle activity associated with pain from muscle activity contributing to physical tooth wear may improve precision in future analyses. Use of STAB may therefore be advantageous, as it captures multiple dimensions of bruxism [23]. Future studies may also benefit from complementary device-based assessments, such as portable surface electromyography, which have become more feasible due to advances in device size and mobility [39–42]. A recent validation study showed relatively low sensitivity, but high specificity for tooth wear alone as an indicator of device-based assessment of bruxism, and that subject-based assessment is a valuable tool to examine presence of bruxism [43].

The correlation between physical tooth wear (abrasion and attrition) and overall tooth wear was near linear, whereas the correlation between erosive wear and overall tooth wear was moderate. This finding aligns with previous Swedish data showing a greater contribution of attrition than erosion to tooth wear [26], and contrasts with views that focus primarily on erosive wear while underestimating the role of physical loading [44, 45]. However, erosive wear is known to be influenced by extrinsic factors such as dietary acid exposure [3, 35, 36], and tooth wear is usually a combination of different factors, both erosive and physical [46]. Future studies should therefore investigate whether dietary and other environmental risk factors show different associations with erosive wear compared with attritional and abrasive tooth wear.

Occlusal support was defined using the Eichner Index, with individuals having fewer than two supportive occlusal zones being excluded from the analysis. This approach was based on evidence demonstrating sharp declines in occlusal force when fewer than two supporting zones are present [47]. Among individuals with adequate occlusal support, the number of remaining teeth was not associated with tooth wear, which may give some support to the validity of the shortened dental arch concept as occlusal forces are maintained [48]. Nevertheless, a strong association, albeit weak correlation, was observed between subjective chewing ability and tooth wear.

Higher BMI showed a weak correlation with tooth wear into dentin. Weight associated factors have primarily been investigated in younger populations, limiting comparability [3]. The observed association to increased tooth wear into dentin and a higher BMI (Table 3) may reflect unmeasured confounding factors, such as gastro-oesophageal reflux disease (GERD) or dietary habits [49, 50]. Evidence from previous studies points to GERD as a large contributing factor in modern erosive tooth wear [51, 52]. Although this study did not take GERD or dietary habits into account, the tooth wear observed in this study was predominantly associated with attritional and abrasive wear rather than erosion. Tooth wear due to GERD can be hard to detect in younger cohorts, as GERD has been shown to not contribute to a significant change of occlusal wear if the condition has been present less than 5 years [50, 52]. GERD has also been reported to co-occur with sleep bruxism; it has been hypothesised that masticatory muscle activity may represent a protective response to reflux episodes, or that the two conditions share common underlying mechanisms [53]. In addition, other potentially important variables, including alcohol consumption and medication use were not available in the dataset [3]. Stress or anxiety is lacking too but was in a scoping review not found to be correlated to tooth wear [3]. These factors may influence tooth wear due to altering oral pH, salivation or tooth grinding patterns, and could therefore have contributed to residual confounding. Smoking was excluded due to its low prevalence in Sweden and previous findings from Jönköping showing no significant association with tooth wear [16, 54].

Examination year was not included as a covariate because the questionnaire, diagnostic criteria, examination protocol, and principal investigator remained unchanged between the two examination periods. By combining the datasets from 2013 and 2023, the sample size increased, improving the precision and robustness of the estimates while reducing the influence of random variation. Although pooling data across survey years may obscure minor temporal changes, the stable oral health status of the Swedish population and the current Swedish dental care reimbursement system, which has been in effect since 2008, suggest that any resulting bias is likely to be minimal [17, 55]. Furthermore, the primary objective was to evaluate individual-level associations with tooth wear rather than temporal trends. This interpretation is further supported by the similarities between the estimates (Table 3 and Additional Table 1). Because salivary variables were only available from the 2013 examination, analyses including these variables effectively reflected that part of the cohort alone. Salivary characteristics were not found to be associated with tooth wear. Previous systematic reviews have identified salivary flow rate as a potential modifier of tooth wear risk, but inclusion of salivary variables in the multivariable analyses had only a minor impact on the results [56]. However, incorporating these variables substantially reduced the sample size and, consequently, the statistical power to detect weaker associations. The lack of significant findings for salivary variables should therefore be interpreted with caution.

Educational level was not associated with tooth wear in this cohort, in contrast to findings from the Netherlands [57]. Sweden’s strong public dental care system and relatively equitable oral health distribution may reduce socioeconomic gradients in oral health outcomes [58], potentially explaining this discrepancy.

Future studies on tooth wear would benefit from formal inter‑examiner reliability assessment and inclusion of additional potential predictors, such as GERD, preferably within a longitudinal design. Repeated assessment of subject-based bruxism over time may help distinguish persistent from transient parafunctional behaviour and reduce exposure misclassification.

Future studies should use longitudinal designs and contemporary multidimensional bruxism assessment methods to determine whether subject-based bruxism is associated with future tooth wear progression. Such studies would also allow evaluation of preventive interventions and the clinical utility of screening approaches.

## Conclusions

Tooth wear in this population was primarily associated with physical rather than erosive wear. Subject-based bruxism showed an independent association with tooth wear that exceeded those observed for age and sex. Incorporating a question of subject-based bruxism behaviour into routine dental examinations may represent a simple and cost-effective strategy to identify individuals with increased levels of tooth wear. Because of the cross-sectional design, the findings should be interpreted as hypothesis-generating and require confirmation in longitudinal studies before their predictive or screening value can be established.

## Supplementary Information

Additional file 1.

## Data Availability

The data that support the findings of this study are available from the Department of Odontology and Oral Health at Jönköping University and The Institute for Postgraduate Dental Education in Jönköping, but restrictions apply to the availability of these data, which were used under license for the current study, and so are not publicly available. Data are however available from the authors upon reasonable request and with permission of the Department of Odontology and Oral Health at Jönköping University and The Institute for Postgraduate Dental Education in Jönköping.

## Abbreviations

BMI: Body mass index
DC/TMD: Diagnostic Criteria for Temporomandibular Disorders
FDI: FDI World Dental Federation General Assembly
GERD: Gastroesophageal reflux disease
GLM: Generalised linear model
HCDI: Helkimo Clinical Dysfunction Index
LR χ^2^: Likelihood‑ratio chi‑square tests
SDA: Shortened dental arch
sEMG: Surface electromyography
STAB: Standardised Tool for the Assessment of Bruxism
TMD: Temporomandibular disorder
