# Supplementary material for: Factors associated with tooth wear in a Swedish adult population: a cross‑sectional study
Source: BMC Oral Health. 2026 Jul 25;26:1358. doi: 10.1186/s12903-026-09417-w (PMC13422280; doi:10.1186/s12903-026-09417-w)
Supplement: Supplementary file 1 — Additional file 1. [file 12903_2026_9417_MOESM1_ESM.docx]

**Additional table 1.** Generalised linear model analysis of included variables, not excluding salivary data.

| **Variable** | **Overall tooth wear** | | **Tooth wear into dentin** | |
| --- | --- | --- | --- | --- |
| *N*=371 | ***P* value** | **Coefficient (95% confidence interval)** | ***P* value** | **Coefficient (95% confidence interval)** |
| Male sex | 0.015 | 0.061 (0.012 – 0.110) | 0.029 | 0.025 (0.003 – 0.048) |
| Higher age | 0.008 | 0.002 (0.001 – 0.003) | 0.135 | 0.001 (0.000 – 0.001) |
| More teeth | 0.635 | 0.002 (-0.006 – 0.010 | 0.185 | -0.002 (-0.006 - 0.001) |
| Higher body mass index | 0.423 | 0.002 (-0.003 – 0.008) | 0.067 | 0.002 (0.000 – 0.005) |
| Subject-based bruxism | <0.001 | 0.149 (0.071 – 0.162) | <0.001 | 0.054 (0.026 to 0.082) |
| Higher Helkimo clinical dysfunction index (reference category 3) | 0.346 | 0: -0.033 (-0.240 – 0.174)  1: -0.078 (-0.282 – 0.126)  2: -0.069 (-0.277 – 0.140) | 0.270 | 0: 0.010 (-0.085 – 0.104)  1: 0.017 (-0.077 – 0.110)  2: -0.014 (-0.110 – 0.082) |
| Higher attained educational level  (reference category academic degree) | 0.641 | CS: -0.032 (-0.101 – 0.036)  SS: -0.018 (-0.073 – 0.038) | 0.906 | CS: 0.003 (-0.028 – 0.034)  SS: 0.006 (-0.020 – 0.031) |
| More regular medical check-ups | 0.094 | -0.017 (-0.073 – 0.040) | 0.596 | 0.013 (-0.013 – 0.039) |
| Hyposalivation | 0.109 | 0.061 (-0.014 – 0.136) | 0.305 | 0.018 (-0.016 – 0.052) |
| Low buffer capacity | 0.632 | -0.012 (-0.063 – 0.038) | 0.220 | -0.014 (-0.038 – 0.009) |
| *P* values were obtained using Type III likelihood‑ratio (LR) χ² tests, representing partial effects of each variable adjusted for all other covariates in the multivariable model. CS = Compulsory school. SS = Secondary school. | | | | |

**Additional table 2.** Pearson correlation between the different types of tooth wear with salivary data included.

| **Degree of tooth wear** *N*=371 | **Classification of tooth wear** | **Pearson correlation** | ***P* value** |
| --- | --- | --- | --- |
| Overall tooth wear | Due to attrition or abrasion | 0.963 | <0.001 |
|  | Due to erosion | 0.396 | <0.001 |
| Tooth wear into dentin | Due to attrition or abrasion into dentin | 0.928 | <0.001 |
|  | Due to erosion into dentin | 0.314 | <0.001 |
